# Rubber Band Training Improves Athletic Performance in Young Female Handball Players

**DOI:** 10.5114/jhk/175396

**Published:** 2024-02-17

**Authors:** Nawel Gaamouri, Mehrez Hammami, Yosser Cherni, Dustin J. Oranchuk, Roland van den Tillaar, Mohamed Souhaiel Chelly

**Affiliations:** 1 Research Laboratory (LR23JS01) Sport Performance, Health & Society, Higher Institute of Sport and Physical Education of Ksar Saîd, University of Manouba, Tunis, Tunisia.; 2 Higher Institute of Sport and Physical Education of Ksar Said, University of Manouba, Tunis, Tunisia.; 3 Muscle Morphology, Mechanics and Performance Laboratory, Department of Physical Medicine and Rehabilitation, University of Colorado Anschutz Medical Campus, Aurora, Colorado, USA.; 4 Department of Sports Sciences and Physical Education, Nord University, Levanger, Norway.

**Keywords:** resistance training, change of direction, standing long jump, repeated sprint ability, force-velocity test

## Abstract

This study's objective was to investigate the impact of a 10-week in season contrast rubber band training program on athletic performance in young female handball players. Youth athletes (15.8 ± 0.2 years) were randomly assigned to an intervention (n = 16) or a control group (n = 14). The intervention group performed contrast rubber band training (20 sessions over two weeks), while the control group maintained regular in-season training. The modified T-test, squat jump, countermovement jump, standing long jump, repeated sprint ability, 1-RM bench press and half squat, along with upper and lower limb force-velocity tests were performed. The intervention group experienced significantly larger performance enhancements than the control group in the modified T-test [p < 0.001; d = 1.45%Δ (intervention = −7.1, control = −0.8)], vertical jump [p ≤ 0.009; d ≥ 0.72; %Δ (8.4 < intervention < 19.8, 4.1 < control < 12.2)], 1-RM strength [p ≤ 0.04, d ≥ 0.80; %Δ (37.1 < intervention < 39.7, 7.2 < control < 11.2)], all force-velocity scores for the upper limbs [p ≤ 0.009; d ≥ 0.72; %Δ (21 < intervention < 82, 0.1 < control < 11.6)], three of four force-velocity scores for the lower limb performance [p ≤ 0.02; d ≥ 0.64; %Δ (6.4 < intervention < 31.3, 0.8 < control < 11.1)] and all repeated sprint times [p < 0.001; d ≥ 1.15; %Δ (−3.4 < intervention < −3.1, −1.9 < control < −0.5)]. It was concluded that ten weeks of contrast rubber band training positively affected most motor abilities in youth female handball athletes. Therefore, coaches and practitioners should consider utilizing contrast rubber band strength training as a time and resource-efficient means of improving physical fitness of youth handball players.

## Introduction

Fast and dynamic actions, including accelerations, hops, throws, changes in direction, and hard body contacts, are typical in female handball. These movements are commonly interspersed with low-intensity ones such as standing and strolling (Luteberget et al., 2018; Michalsik et al., 2015; Wagner et al., 2018; Wik et al., 2017). Maximum strength, power and its derivatives (jumps, changes of direction, and repeated sprints) are among the physical requirements for success in handball (Luteberget and Spencer, 2017; Luteberget et al., 2018). Thus, strength and conditioning programs should consider these physical requirements (Abade et al., 2019; Hammami et al., 2019a; Hermassi et al., 2010; Mascarin et al., 2017a). However, while strength and conditioning professionals should frequently implement strength and power training strategies, access to equipment is often lacking.

Compared to traditional resistance training equipment, rubber band resistance training equipment is less costly, more portable, and simpler to incorporate into regular handball training sessions (Aloui et al., 2019a; Andersen et al., 2018; Mascarin et al., 2017b). Most research investigating the long-term effects of rubber band exercise on strength and power, sprint, and athletic performance has found positive results (Andersen et al., 2018; Lopes et al., 2019; Mascarin et al., 2017b; Oranchuk et al., 2021). Young female handball players' performance in the countermovement jump with or without an arm swing, power output at lighter loads, three throwing velocity tests, and repeated agility runs improved more over nine weeks of rubber resistance band training (three times per week) than it did throughout the control period, according to research by Andersen et al. (2018). Similarly, Mascarin et al. (2017b) found increases in average internal shoulder rotation power and throwing velocity after six weeks (three times per week) in young female handball players following rubber band focused strength and conditioning training.

Contrast rubber band training has received less attention in the literature than contrast training, although contrast training has been previously studied regarding team-sport performance measurements (Cormier et al., 2020). Several studies have examined how contrast training affects adults' and youth athletes' physical performance, and have demonstrated improvements in sprint, jump, and change of direction test results (Cormier et al., 2020; Elbadry et al., 2019; Hammami et al., 2017, 2019b). Contrast training aims to develop strength and neuromuscular power by combining high- and low-load exercises in the same workout (Smilios et al., 2005). Cometti (1998) defined this method as six repetition sets with 70–90% of 1 repetition maximum (1-RM) loads, alternated with six repetition sets with loads between 30 and 50% 1-RM, executed at maximum speed. Conversely, relatively few interventions have evaluated the effects of strength training using rubber bands (i.e., strength rubber bands, plyometric training with rubber bands, resistance training with rubber bands) (Aloui et al., 2019a, 2019b; Andersen et al., 2018; Mascarin et al., 2017b).

However, no previous studies have examined the effects of contrast rubber band exercise on athletic performance in female youth athletes. Therefore, this research sought to determine the effects of contrast rubber band exercises (i.e., six repetitions of high-load followed by six repetitions of low–load exercises) on athletic performance in U17 woman handball athletes. We hypothesized that the ten-week, biweekly, contrast rubber band training program would enhance the performance measures relevant to handball athletes.

## Methods

### Participants

Thirty young female national-level handball players from two clubs were recruited to participate. A sample size of thirty was determined via the G power 3.0.10 program with the following criteria: Z1-β = 0.84 (power = 80%) and Z/2 = 2.58 (alpha = 1%). According to Meszler and Váczi (2019), the countermovement vertical jump's mean and standard deviation were 33.5 ± 3.9 cm and 28.7 ± 6.7 cm, respectively, in the experimental group and 14 experimental and 14 control individuals, respectively, were required (Faul et al., 2007). Players from each position were divided according to their playing position and randomly allocated to either the intervention (n = 16) or the control group (n = 14) (Table 1). Their mean experience of handball competition was at least five years, with at least three years’ experience in strength training. All had already achieved excellent overall physical preparation at the beginning of the season (a preliminary 6-week period of six training sessions per week). This initial phase was divided into two parts. During the first three weeks, a resistance training program aimed to improve muscular power and muscle hypertrophy with light loads (40–60%, one repetition maximum) was conducted. The second 3-week period was devoted to improving muscular strength with higher loads (70–85%, one repetition maximum), supplemented with participation in friendly matches each weekend. Participants continued to engage in five handball sessions per week during September when the competitive period began.

**Table 1:** Physical characteristics of the intervention and control groups (mean ± SD).

	Intervention group (n = 16)	Control group (n = 14)
Age (years)	15.80 ± 0.30	15.80 ± 0.20
Body mass (kg)	64.10 ± 3.50	63.40 ± 4.10
Body height (m)	1.67 ± 0.34	1.67 ± 0.32
% Body fat	22,00 ± 2.70	22.70 ± 1.90
Age of peak height velocity (years)	12.70 ± 0.40	12.90 ± 0.30
Predicted years from APHV (years)	3,00 ± 0.40	2.90 ± 0.40

APHV: Age of peak height velocity

**Figure 1:**
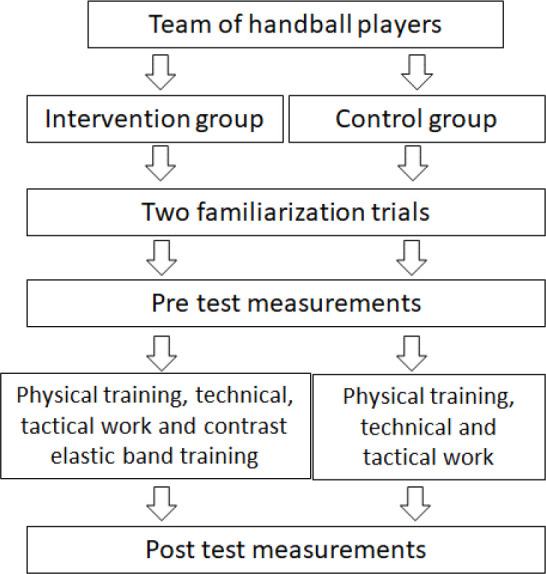
Intervention set up.

Athletes had five years of experience in handball training with an average of 4–5 sessions per week (~1–2 h each session). They played an official or a friendly match every Saturday or Sunday. During the 10-week intervention, both groups followed their regular workout regimens. Contrast rubber band training was substituted for some of the intervention group's technical/tactical sessions (Figure 1).

To reduce bias, the researchers who analyzed the data were separate from the researchers who collected the data. By the guidelines outlined in the most recent version of the Declaration of Helsinki, written informed consent was obtained from the athletes and their parents or guardians before participation. This study was approved by the local ethics committee of the Higher Institute of Sport and Physical Education of Ksar Saîd (UR17JS01; October 2018).

### Design and Procedures

The study was designed to test the effects of a 10-week contrast rubber band training program on selected fitness measures in youth female handball players. The experimental intervention of biweekly contrast rubber band training was undertaken during the second phase of the national championships (January to March). All participants had previously engaged in five to six weekly training sessions (90–120 min each). However, for ten weeks, the intervention was not added to regular handball training. Still, it was immediately performed after the warm-up program, replacing some low-intensity technical-tactical handball drills. The intervention replacement activity accounted for < 10% of the total handball-training load (competitive and friendly matches not accounted for). At the same time, the control group carried out technical exercises such as passing, shooting, etc. The control group followed their regular handball training (i.e., mainly technical-tactical drills, small-sided and simulated games, and injury prevention drills). The overall handball training load was comparable between groups (using the Borg Rating of Perceived Exertion (RPE)) as they followed similar handball training routines consisting of 5 sessions of 90–120 min per week.

### Measures

All measurements were taken four days after the final intervention session and one week before the experiment's start. The tests were completed on five separate days. Day 1 included anthropometry and lower limb force-velocity tests; day 2: jump tests (squats, countermovement and standing long jumps) and the 1-RM bench press; day 3: the modified agility T-test and the 1-RM half squat; day 4: repeated sprint ability; and day 5: the upper limb force-velocity test. In the week before the intervention started, two sessions were conducted to evaluate the reliability of change of direction (the modified agility T-test) and jump (squat, countermovement and standing long jumps) tests. All participants were re-familiarized with testing procedures one day after the final contrast rubber band training session.

#### Anthropometric Measurements

A single investigator carried out all anthropometric measurements, including body height, sitting height, body mass, body fat content and biological age (Durnin and Womersley, 1974; Mirwald et al., 2002).

#### Modified Agility T-Test

As recommended by Haj-Sassi et al. (2011), the modified T-test was employed to record speed with directional changes (using paired photocells from Microgate, Bolzano, Italy).

#### Vertical Jumps

The Optojump System (Microgate SARL, Bolozano, Italy) was used to measure jump height. Under researcher supervision, participants started the squat jump with their knees bent at 90° and holding this position for 3 s before maximally completing each jump. The countermovement jump started with participants standing straight up, quickly bending their knees to 90° before rapidly changing the direction and jumping vertically. Both squat and countermovement jumps were performed with the hands placed on hips.

#### Standing Long Jump

Participants stood with their legs bent and arms back. Participants counter-moved with their arms and legs as soon as the command of “ready, set, go!” was given before leaping as far as possible. Participants could not fall forward or backward, and had to land with both feet simultaneously. We measured the horizontal distance to the closest millimetre between the starting line and the heel of the back foot with a tape measure.

#### Repeated Sprint Ability (RSA) Test

The repeated sprint ability test consisted of six sets of two 20-m round trip sprints using a paired photocell. Each sprint started 20 s apart (Buchheit et al., 2010). The best and total sprint times were recorded, and the fatigue index was calculated (Buchheit et al., 2010).

#### 1-RM Half Squat and the Bench Press

Participants performed several sets (up to six sets, with four minutes of passive rest in between) while gradually increasing the loads (5–10% of the self-reported 1-RM for the bench press and 10–20% of the self-reported 1-RM for the half squat); the maximum possible load was completed (Grgic et al., 2020).

#### Force-Velocity Test

A Sweden Monark, model 894 E was used for this test, to determine the strength of the limbs during intense short pedaling sprints (maximum 7 s) against loads of 1.5%, 2%, 2.5%, 3%, 4.5%, and 5% of body mass for the upper limbs, and 7.5%, 8.5%, 9.5%, 10.5%, and 11.5% of body mass for the lower limbs. Peak power (W), peak power per kilogram of body mass (W/kg), maximum pedaling velocity (rpm), and maximum braking force (N) were calculated (Chelly et al., 2010).

### Training Program

The training program included a progressive 10-week training program using contrast rubber bands (Table 2). Eight exercises (numbered from one to eight, four for the upper and the lower limbs, respectively) were performed during the biweekly sessions (Tuesdays and Thursdays, a total of 20 sessions). The lower limb exercises (knee extension, knee flexion, half-squats, and hip adduction) are represented by even numbers, and the upper limb activities are represented by odd numbers (flies, rows with high elbows, trunk rotation, and standing presses). Thera-Bands are a rubber band system of four latex bands: red, green, blue, and black. The length and the load of each rubber band are reported in Table 2. Our training program was similar to that of Hammami et al. (2022). Training sessions were preceded by a 15-min warm-up and lasted for 30 min (a total of 45 min).

**Table 2:** Contrast rubber band training program.

Exercises	Week 1		Week 2	Week 3		Week 4			Week 5		
Load	Sets × Reps										
*Upper limb*										
Resistance at 250 and 100% elongation	Red rubber band (3.2 and 1.8 kg)		Green rubber band (4.4 and 2.3 kg)						Blue rubber band (6 and 3.2 kg)		
Flies, row with high elbows, trunk rotation and standing press	3 × (6+6)		3 × (6+6)	4 × (6+6)		5 × (6+6)			3 × (6 + 6)		
*Lower limb*										
Resistance at 250 and 100% elongation	Red rubber band folded (6.4 and 3.6 kg)		Green rubber band (8.8 kg and 4.6 kg)						Blue rubber band folded (12 and 6.4 kg)		
Knee extension and flexion, half squat and hip adduction	3 × (6+6)		3 × (6+6)	4 × (6+6)		5 × (6+6)			3 × (6+6)		
	**Week 6**	**Week 7**			**Week 8**		**Week 9**				**Week 10**
*Upper limb*										
Resistance at 250 and 100% elongation	Blue rubber band (6 and 3.2 kg)			Black rubber band (8 and 4.4 kg)							
Flies, row with high elbows, trunk rotation and standing press	4 × (6+6)	5 × (6+6)		3 × (6+6)				4 × (6+6)		5 × (6+6)	
*Lower limb*										
Resistance at 250 and 100% elongation	Blue rubber band folded (12 and 6.4 kg)			Black rubber band folded (16 and 8.8 kg)							
Knee extension and flexion, half squat and hip adduction	4 × (6+6)	5 × (6+6)		3 × (6+6)				4 × (6+6)		5 × (6+6)	

Participants performed first 6 reps at 250% elongation of the initial length of the rubber band directly followed by 6 repetitions at 100% elongation.

### Statistical Analysis

The Windows version of SPSS 22 was used to conduct statistical analysis (SPSS, Inc., Armonk, NY: IBM Corp). The Kolmogorov-Smirnov test was used to examine the normality of all variables. The data are displayed as a mean and a standard deviation (SD). Independent *t*-tests were used to investigate between-group differences at baseline, and two-way analyses of variance (intervention vs. control and pre- vs. post-test) were used to determine the impact of the intervention. Paired sample *t*-tests were used to assess within-group pre-to-post performance improvements. Cohen's *d* effect sizes were categorized as small (0.00 ≤ *d* ≤ 0.49), medium (0.50 ≤ *d* ≤ 0.79), and large (*d* ≥ 0.80) (Cohen, 1988). The threshold for statistical significance was established at *p* < 0.05. Two-way mixed intraclass correlation coefficients (ICCs) and coefficients of variation (CVs) were used to evaluate the dependability of all dependent variables.

## Results

ICC values were between 0.91 and 0.98, and CV values between 2.2 and 10% for all the variables (Table 3).

**Table 3:** Reliability and variability of performance tests.

	Intraclass correlation coefficient	95% confidence intervals	Coefficient of variation (%)
T-half test	0.916	0.893–0.930	2.2
Standing long jump	0.990	0.978–0.995	10
Squat jump	0.979	0.956–0.990	10
Countermovement jump	0.987	0.972–0.994	9

**Figure 2:**
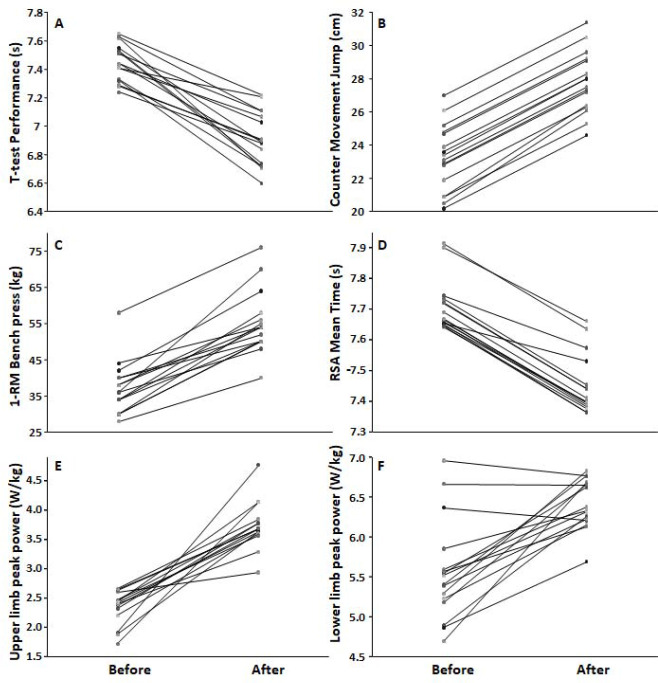
Spaghetti graph of (A) the modified agility T-test, (B) the counter movement jump, (C) the 1-RM bench press, (D) repeated sprint ability (RSA) mean time, (E) upper limb peak power, and (F) lower limb peak power before and after an 8-week intervention including the contrast rubber band training program.

With a group×time interaction, the intervention group experienced greater enhancements in the T-half performance (*p* < 0.001; *d* = 1.45) (Figure 2-A), vertical jump performance (*p* ≤ 0.009, *d* ≥ 0.72) (Figure 2-B), maximal 1-RM bench press strength (*p* ≤ 0.04, *d* ≥ 0.80) (Figure 2-C), repeated sprint times (*p* < 0.001, *d* ≥ 1.15) (Figure 2-D), all force-velocity variables for the upper limbs (*p* ≤ 0.009, *d* ≥ 0.72) (Figure 2-E) and the peak power and braking force variables for the lower limbs (*p* ≤ 0.02, *d* ≥ 0.64, Table 4) when compared to the control group (Figure 2-F).

No significant between-group differences were noted for the horizontal jump (*p* = 0.422, *d* = 0.22), the fatigue index (*p* = 0.75, *d* = 0.08), and maximal lower limb pedaling velocity (*p* = 0.194, *d* = 0.35) improvements (Table 4).

**Table 4:** Means and standard deviations for all considered variables at baseline and following the intervention in the experimental and the control group

	Intervention group (n = 16)				Control group (n = 14)				Interaction
	Pre	Post	% change	Cohen’s *d*	Pre	Post	% change	Cohen’s *d*	Cohen’s *d*
***Change of direction***									
T-test (s)	7.45±0.13	6.92±0.16	−7.1±2.7	3.76*	7.49±0.16	7.42±0.18	−0.8±0.8	0.43*	1.45*
***Jump***									
Squat jump (cm)	22.9±2	26.9±2.3	17.4±3.7	−1.92*	22.7±2.3	23.7±1.8	4.7±4.6	−0.50*	0.72*
Countermovement jump (cm)	23.2±2	27.8±1.9	19.8±3.1	−2.44*	23.9±2.2	24.9±1.9	4.1±3.5	−0.50*	0.93*
Standing long jump (m)	1.48±0.10	1.60±0.10	8.4±6.2	−1.24*	1.52±0.15	1.69±0.17	12.2±13	−1.10*	0.22
***Repeated sprint ability***									
Best time (s)	7.54±0.10	7.29±0.11	−3.3±0.7	2.46*	7.54±0.07	7.50±0.06	−0.5±0.4	0.39*	1.15*
Total time (s)	46±0.5	44.4±0.6	−3.4±0.6	2.99*	46.2±0.4	46±0.4	−0.5±0.5	0.52*	1.38*
Fatigue index (%)	1.63±0.39	1.55±0.37	−3.1±17.4	0.22*	2.16±0.60	2.16±0.55	−1.9±19.5	0.04	0.08
***1-Repetition maximum***									
Bench press (kg)	37±7.3	55.1±8.7	39.7±26.7	−1.65*	35.3±10.4	39±10.7	11.2±5.6	−0.36*	0.80*
Half squat (kg)	78.9±9.5	107.1±8.7	37.1±15.1	−3.20*	76.6±9.2	82±9.5	7.2±3.4	−0.60*	1.17*
***Upper limb***									
Peak power (W)	150±18.3	268.1±26.3	82±34.9	−5.38*	146±24	146.5±10.7	2.7±17.6	−0.03	2.90*
Peak power per body mass (W/kg)	2.33±0.29	3.72±0.41	63.1±36.2	−4.04*	1.88±0.25	1.92±0.22	3.8±18.7	−0.18	2.28*
Maximal pedalling velocity (rpm)	89.7±16.5	105.6±9.8	21±23	−1.21*	87.3±17.3	84.2±8.6	0.1±21.5	0.24	0.72*
Maximal braking force (N)	6.89±1.11	9.86±1.28	46.1±26.9	−2.56*	6.53±1.16	7.09±0.73	11.6±23.9	−0.06	1.11*
***Lower limb***									
Peak power (W)	357.5±25.5	424.5±29	20.3±13.7	−2.53*	337.8±37.9	355.4±35.9	5.3±3	−0.49*	0.64*
Peak power per body mass (W/kg)	5.57±0.63	6.42±0.31	16.5±12.5	−1.77*	5.28±0.55	5.45±0.46	3.3±3.3	−0.35*	0.71*
Maximal pedalling velocity (rpm)	169±20.3	157±15.7	6.4±10.5	0.68*	162.9±18.8	164.1±22.4	0.8±8.8	−0.06	0.35
Maximal braking force (N)	8.02±0.84	10.5±0.70	31.3±12.8	−3.25*	7.83±0.51	8.69±1.07	11.1±12.8	−1.06*	1.00*

* indicates a significant effect at a *p* < 0.05 level

## Discussion

The present research aimed to assess the efficacy of a ten-week contrast rubber band training to improve change of direction ability, jumping, maximal strength, peak power output, and repeated sprint ability scores for youth female handball players. The contrast rubber band training group experienced significantly greater improvements than the control group, with the exception of the horizontal jump (i.e., standing long jump), the fatigue index and maximal pedalling velocity of the lower limbs. Therefore, the affordable and portable contrast rubber band training program should be considered by strength and conditioning specialists when working with youth female handball players, especially in- season.

The present study's findings showed more significant gains in change of direction (T-half) performance in the intervention compared to the control group, which was in line with Cormier et al.'s (2020) meta-analysis that reported a medium effect (*d* = 0.68) after contrast training on change of direction performance in team sport athletes. On the contrary, young female handball players who underwent 6-week strength training with rubber bands showed no significant improvement in agility, according to Andersen et al. (2018)(Vidar Andersen et al., 2018). The age of the study population, the type, intensity, duration, the number of training blocks, and timing of the studies around the playing season may all be contributing factors to dichotomous study finding. In this regard, Pardos-Mainer et al. (2021) demonstrated that strength training interventions were more effective with longer study duration (> eight weeks), a higher number of training sessions (> 16), and greater session length (> 30 min). Larger improvements in muscle strength were also seemingly responsible for improving change of direction performance. Therefore, the rubber band-tension loading variables may enable higher force output via a more comprehensive movement range and faster contractions due to extended force application periods (Katushabe and Kramer, 2020; Wisløff et al., 2004).

All vertical jump performances improved significantly in the intervention group compared with the control group, which can be attributed to neural adaptation (Kraemer et al., 1996; Sale, 1988). This hypothesis is partially supported by Labib (2013), who reported increased standing long jump and jump shot performance after complex training in female handball players between 18 and 22 years. Another possible explanation for contrast training-induced vertical jump enhancement may be linked to greater total training volumes in contrast training than in other interventions, which may, in turn, have produced greater neuromuscular stimulation (Wilson et al., 2013). Conversely, standing long jump changes did not differ between groups. This may be due to the fact that coordinative abilities, rather than strength or neuromuscular factors, are arguably more important for horizontal jumps.

Handball performance depends not only on strength, but also on the ability to exert force at sport specific speeds (Luteberget and Spencer, 2017; Luteberget et al., 2018; Manchado et al., 2013). Powerful actions are often associated with high-velocity movements (Luteberget and Spencer, 2017; Luteberget et al., 2018; Manchado et al., 2013). The present study demonstrated that contrast rubber band training increased both 1-RM performances. The review by Lopes et al. (2019) showed that rubber resistance exercises increased muscle power. Despite no change in body mass, most investigations found that absolute strength improved (Aloui et al., 2019a, 2019b; Hermassi et al., 2019). Given that both neurological and morphological adaptations can be used to explain changes in strength (Folland and Williams, 2007), changes in intra- and inter-muscular coordination are likely to underpin gains. Additionally, it is understood that individuals who have not been resistance trained see early strength increases due to neural, rather than structural adaptations (Kraemer et al., 1996). Those with little to no experience with strength training may anticipate an improvement in their capacity to produce force (Cormier et al., 2020; Sale, 1988). However, concurrent training regimens have repeatedly been found to reduce strength dependent adaptions (Wilson et al., 2012).

Our study showed moderate to large improvements in limb power in the intervention group against the controls which is highly relevant to handball performance (Saavedra et al., 2018). However, because of methodological differences, it is not easy to compare our results with previous research. To the authors’ knowledge, no study has previously addressed the effects of contrast rubber band training on force-velocity performance in young female athletes. Similar studies in boys have shown that cluster sets with rubber bands improve performance of the force-velocity test. In line, Aloui et al. (2019b) reported more significant improvements in absolute (49.3 ± 22.9%) and relative peak power (47.9 ± 24.6%) following an eight-week bi-weekly upper limb rubber band training program in U19 male handball players, while relative peak power and maximal pedalling velocity of the lower limbs remained unchanged. Also, Hermassi et al. (2019) found substantial power enhancement in both the upper (31.3%; *p* < 0.001) and lower (17.5%; *p* < 0.001) limbs after 12 weeks of weightlifting training in male handball players. In brief, the present study showed that contrast rubber band training was an equally effective training intervention in improving young female handball players’ force-velocity performance. Factors that govern power improvement following contrast rubber band training are essentially of the neuronal origin, including enhanced muscle activation techniques (i.e., inter-muscular coordination) and improved motor unit recruitment (i.e., intra-muscular coordination) (Markovic and Mikulic, 2010; Stojanovic et al., 2017).

Regarding repeated sprint ability, the current investigation showed that the intervention group experienced large improvements in this domain, which is supported by similar studies in boys' handball athletes. For example, Aloui et al. (2020) discovered that incorporating eight weeks (with two sessions per week) of lower limb plyometric training using rubber bands into the in-season program improved repeated change of directions scores in junior handball players. Likewise, Aloui et al. (2019a) revealed improvements in two of four repeated change of direction scores in junior male handball players. The mechanism responsible for this effect has been attributed mainly to improvements in anaerobic fitness and muscle buffer capacity that could encourage quicker phosphocreatine resynthesis (Bishop and Spencer, 2004; Bishop et al., 2001).

While the primary aims of the present study were completed, there are several limitations to consider. Firstly, we did not include any direct neuromuscular evaluations, such as electromyography or isokinetic strength evaluations. Similarly, we did not assess changes in muscle size, making it difficult to understand the relationships between morphological and performance adaptations. Readers should also be cognisant that the present findings may not be applicable to other sports, age groups, performance levels or sexes. Additionally, exercise selection, training frequency, duration and progression, and its timing relative to the competitive season are all important factors. Future studies can build upon these limitations to further understand contrast rubber band training.

## Conclusions

The present study shows that the replacement of part of the usual handball training by contrast rubber band training (< 10% of the total handball-training load) in the youth female players' standard handball training regimen for ten weeks is sufficient to improve markers of handball-related athletic performance. Considering this, adding contrast rubber band training to handball teams' regular training regimens may be advantageous and practical, especially when time and traditional resistance training equipment are not available or limited.
